# Immediate effects of temporary bite-raising with light-cured orthodontic band cement on the electromyographic response of masticatory muscles

**DOI:** 10.1590/1678-7757-2017-0214

**Published:** 2018-05-03

**Authors:** Darin Pativetpinyo, Weera Supronsinchai, Chidsanu Changsiripun

**Affiliations:** 1 Faculty of Dentistry, Department of Orthodontics, Chulalongkorn University, Bangkok, Thailand; 2 Faculty of Dentistry, Department of Physiology, Chulalongkorn University, Bangkok, Thailand

**Keywords:** Electromyography, Masticatory muscles, Vertical dimension, Orthodontics

## Abstract

**Objective:**

To assess the immediate effects of temporary bite-raising using light-cured orthodontic band cement on the superficial masseter and anterior temporalis electromyography (EMG) activity in healthy adults.

**Materials and Methods:**

Surface EMG signals were recorded bilaterally from the superficial masseter and anterior temporalis muscles of 30 volunteers with a normal occlusion, before and after having temporary bite-raising. The bite-raising was done by adding light-cured orthodontic band cement (3x5x2 mm WxLxH) on the lingual cusps of both upper first molars. The measurements were recorded (i) at rest, (ii) while clenching in centric occluding position and (iii) while chewing on an artificial test food. The EMG activity at rest and during clenching, the maximum voltage, and the duration of the identified EMG signal burst while chewing the artificial test food before and after temporary bite-raising were statistically compared using the paired t-test or the Wilcoxon signed-rank test based on the normality of the variables. The significance level was set at 5%.

**Results:**

After temporary bite-raising, we found no significant change in integral EMG activity at rest position for the superficial masseter (mean difference (MD)=7.5 μVs) and for the anterior temporalis muscle (MD=36.8 μVs); however, the integral EMG activity during clenching was significantly reduced for the superficial masseter (MD=201.2 μVs) and for the anterior temporalis muscle (MD=151.8 μVs). During mastication, the maximum voltage of the identified burst was significantly reduced on the preferred chewing side of the superficial masseter and anterior temporalis muscles (MD=127.9 and 47.7 μV, respectively), while no significant change was found for the duration of the identified burst (MD=-34.1 and 3.4 ms, respectively) after temporary bite-raising.

**Conclusion:**

The results point to an altered neuromuscular behavior during clenching and chewing immediately after temporary bite-raising with light-cured orthodontic band cement. This information is relevant for orthodontists to inform their patients what will happen to their masticatory muscle activity when this bite-raising method is used.

## Introduction

Temporary bite-raising or bite-opening is often needed in patients undergoing orthodontic treatment, such as in deep overbite and/or crossbite cases. Bite-raising is used to prevent the brackets from being sheared off, and to eliminate occlusal interferences, allowing unobstructed tooth movement^18^. Moreover, bite-raising allows the proper positioning of the brackets during the early treatment phase by keeping specific groups of teeth out of occlusion and preventing full jaw closure^14^. One method of performing bite-raising is adding a light-cured orthodontic band cement to the occlusal surfaces of the posterior molars or lingual surfaces of the anterior teeth^1^ ^,^ ^16^. This procedure is hygienic, minimizes bulkiness, reduces interference with speech, and is less intrusive on the tongue space compared to a conventional removable bite plate. In addition, when bite-raising is used in patients with multi-brackets on every tooth, the orthodontic tooth movement is done without interference from the acrylic plate, and is easy to place on the tooth surfaces in one visit. Build-ups for posterior teeth using this method were recently reported to be an effective alternative treatment for anterior open bite in adults^23^.

Several studies indicate that increased occlusal jaw opening may lead to changes on the electromyographic (EMG) activity of the jaw muscles and there are several studies on the consequence of bite-raising by other means on the EMG activity^4^ ^,^ ^11^. However, we do not know of any published studies on the effects of opening the bite by adding light-cured orthodontic band cement on the occlusal surfaces of the posterior teeth, on jaw muscle activity. Despite the increase on the vertical dimension of this method being similar to other methods, it results in only two occlusal contact areas. This might affect the EMG activity of the jaw muscles differently.

The objective of this study was to examine the immediate masseter and temporalis muscle EMG activity before and after opening the bite with light-cured orthodontic band cement on the lingual cusps of both upper first molars during physiologic rest position, maximum voluntary clenching (MVC), and chewing on artificial test food in normal occlusion participants. The null hypothesis was that bite-raising using this method does not immediately affect the masseter or temporalis muscle EMG activity at rest, during MVC, and chewing.

## Materials and methods

### Participants

Forty-two volunteers, randomly recruited from Dentistry students of the Chulalongkorn University, received a clinical examination. Twelve volunteers were excluded due to having an edentulous area (n=2), overjet >3 mm (n=4), overbite >3 mm (n=5), or open bite (n=1). Thus, 30 volunteers (11 men, 19 women; age range from 18–25 years) participated in this study. The participants were dentate with a Class I occlusion, an overjet less than 3 mm, and an overbite less than 3 mm (mean overbite 2.35±0.65 mm, mean ± S.D.). The participants had no edentulous area, no significant facial deformity, and no temporomandibular symptoms based on the assessment protocol of Schiffman, et al.^19^ (2016). The participants had no previous orthodontic treatment in the past 3 years. No participant missed an appointment or had dental treatment during the experimental period. The study protocol was approved by the Human Research Ethics Committee of the Faculty of Dentistry, Chulalongkorn University (HREC-DCU 2015-090). All participants signed informed consent forms.

### Protocol for measuring muscle activity

The EMG measurements were performed on two appointments, with a week interval between each. The objective of the study and the measurement procedure were explained to the participants on the first appointment, we also measured the EMG activity without any intervention.

**Figure 1:**
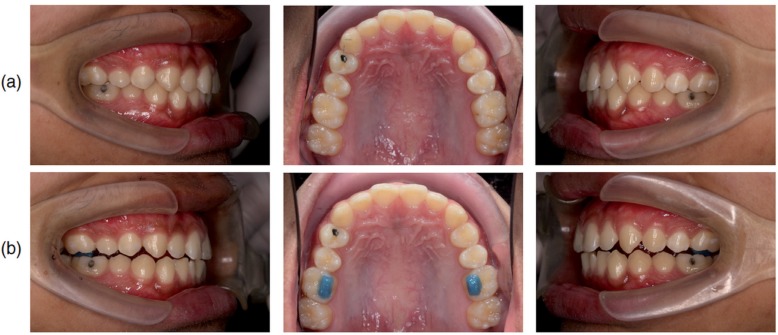
Intraoral photos (a) before and (b) after temporary bite-raising with light-cured orthodontic band cement

During the second appointment, we performed a baseline recording at rest and during MVC. The bite of the participant was then temporarily raised by placing light-cured orthodontic band cement on the lingual cusp of the upper first molars. After polishing, rinsing, drying, and isolating the teeth, Ultra Band-Lok BLUE (Reliance Orthodontic Products, Inc., Itasca, IL) was applied in a thermoplastic sheet template (3x5x2 mm WxLxH trapezoid-shaped slot) and placed on the tooth. We used a light-curing unit (600 mW continuous output) to cure the material. The occlusion was balanced by identifying an occlusal contact on each side with shimstock foil and locating the specific contact with the eight-micron thick articulating film according to the technique described by Anderson, Schulte and Aeppli^3^ (1993). Representative intraoral photographs before and after temporary bite-raising are shown in Figure 1. The mean overbite after temporary bite-raising was −0.57±0.70 mm (mean± S.D.). Consequently, the mean interincisal distance change was 2.90±0.20 mm (mean ± S.D.). After the EMG recordings, the bite-raising material was removed using adhesive removing pliers.

### Surface EMG recordings

Surface EMGs were simultaneously recorded from the left and right superficial masseter and anterior temporalis muscles by a researcher using the ML866 PowerLab 4/35 (ADInstruments Pty Ltd., Bella Vista, Australia), following the protocol indicated by the manufacturer. The participants were seated upright on a chair with their trunk perpendicular to the floor, both feet on the floor, hands resting on their lap, and looking forward with their head unsupported in a quiet place listening to relaxing music^20^. We cleaned the skin with isopropyl alcohol (70%), and gently applied a small amount of abrasive gel (ADInstruments Pty Ltd.) on it, which lowered the electrode-skin impedance at the electrode site with minimal discomfort or irritation. Self-adhesive disposable ECG electrodes (ADInstruments Pty Ltd.) were placed bilaterally on the skin of the subject overlying the muscles and fixed with Nexcare Sensitive Skin Tape (3M, St. Paul, MN). For the anterior temporalis muscles, the electrodes were placed 1.5–2 cm posterior to the lateral canthus of the eye, slightly above the zygomatic arch. For the superficial masseter muscles, the electrodes were placed on the midpoint of the bisection of the anteroposterior and inferosuperior dimensions of the muscle belly, which was determined by asking the subject to clench the teeth according to the method described by Teenier, Throckmorton and Ellis^21^ (1991). The inter-electrode distances were recorded for each subject for the left and right sides and used to place the electrode in the latter phase. A ground electrode was placed on the sternal end of the clavicle. External noise was controlled to avoid artifacts caused by smiling or other facial expressions.

After placing the electrodes and practicing for the measurement, the subjects were given a five minutes rest period to relax and for the skin to absorb the conductive gel. The EMG recordings were then made as follows:

### Test A - physiological rest position

The purpose of this test was to monitor and quantify the amount of electrical activity generated by the superficial masseter and anterior temporalis muscles when at rest.

### Test B - maximum voluntary clench (MVC)

The subjects were instructed to clench their teeth in centric occluding position using as much force as possible without causing pain and to hold this force intensity until instructed to relax after 2 seconds.

### Test C - mastication

The subjects were instructed to chew an artificial test food for 20 strokes on their preferred chewing side. When the subjects were not sure of their preferred chewing side, they were instructed to use the right side. The artificial test food was made with a dental impression material, OptoSil (Heraeus Kulzer GmbH, Hanau, Germany), using the standardized production of an artificial test food protocol^2^ and cut into four quarters.

We performed two experimental sessions as recommended in a previous study^8^, the first session was for the participants to become used to the EMG measurement procedure and the second session was for data collection. The data obtained in the second session were used for analysis. For test A and test B, the measurements were repeated three times and each recording took 6–8 seconds for test A and 2 seconds for test B followed by a one minute rest period^21^. For test C, the measurement was done while chewing the artificial test food.

The digital signal was analyzed using LabChart software (ADInstruments Pty Ltd.). For test A and B, the area of the integral EMG curve (duration x mean voltage, μVs), was computed for each muscle. The left and right sides values were pooled for each muscle. The mean integral EMG activity of the three trials for each muscle was expressed as mean and standard deviation. To normalize the EMG signals, the root-mean-square (RMS) value of the EMG signals during the 2 seconds window was calculated and expressed as the percentage of the EMG activity during MVC in centric occlusion.

For test C, the signal was full-wave rectified and smoothed by taking the RMS for each 100 ms interval. The start and the end of each burst were defined as the EMG signal reaching a level 10% above or below the mean area relative to the baseline signal^9^. The maximum voltage (μV) and duration (ms) of the identified burst were computed for each muscle. The first chewing cycle was excluded from the statistical analysis since research suggests that subjects use the first chewing cycle to gather information about an item^15^. The mean of cycles 2–5 were used for statistical comparisons.

### Intra-examiner reliability

The baseline recordings for both muscles at rest were used to evaluate intra-examiner reliability, they were conducted in two visits, with a week interval between them. Both recordings were performed by the same examiner and in the same period of the day, according to the procedure mentioned above.

### Statistical analysis

Statistical analysis was performed using the software SPSS version 17.00 (SPSS Inc., Chicago, IL, USA). The normality of the variables was verified by the Kolmogorov-Smirnov test. For comparisons between before and after bite-raising, the paired t-test or the Wilcoxon signed-rank test was used based on the normality of the data. We tested 16 pairs of before-after data. The paired t-test was performed for the integral superficial masseter and anterior temporalis muscles EMG activity during MVC, the normalized superficial masseter muscle EMG activity during MVC, the maximum voltage of the identified burst on the superficial masseter preferred chewing side, the duration of the identified burst on the superficial masseter and anterior temporalis preferred chewing side, and the duration of the identified burst on the anterior temporalis non-chewing side. The Wilcoxon signed-rank test was performed on the integral superficial masseter and anterior temporalis muscles EMG activity during rest, the normalized superficial masseter and anterior temporalis muscles EMG activity during rest, the normalized anterior temporalis muscle EMG activity during MVC, the maximum voltage of the identified burst on the superficial masseter non-chewing side, the maximum voltage of the identified burst on the anterior temporalis preferred chewing side and non-chewing side, and the duration of the identified burst on the superficial masseter non-chewing side. Significant differences were defined as p<0.05. The effect size was calculated for the significant comparisons [(effect size=SD(Δ)*δ/✓N; α=0.05, β=0.20, N=Sample size, SD(Δ)=Standard Deviation of the change in the outcome, the standard T value (with degrees of freedom) corresponding to α=2.05 and parameter (δ)=2.90)]^17^. Intra-examiner reliability between the two visits was evaluated by intraclass correlation. The intraclass correlation coefficient (ICC) was calculated based on a single rating, absolute-agreement, 2-way mixed-effects model. ICC values <0.5 indicate poor reliability, values between 0.5–0.75 indicate moderate reliability, values between 0.75–0.9 indicate good reliability, and values >0.9 indicate excellent reliability^12^.

## Results

The intraclass correlation coefficient for the superficial masseter and anterior temporalis muscles measurements were 0.93 and 0.91, respectively, indicating an excellent reproducibility of the EMG measurements. No significant differences were found between the superficial masseter integral EMG activity or normalized EMG activity before and after temporary bite-raising at rest. Similar results were also found for the anterior temporalis muscle at rest. However, integral EMG activity and normalized EMG activity for both muscles was significantly reduced during clenching after temporary bite-raising (Table 1 and 2). The effect size of the integral and normalized EMG activity for the superficial masseter muscle was 114.0 μVs and 16.7%, respectively. The effect size of the integral and normalized EMG activity for the anterior temporalis muscle was 120.8 μVs and 21.4%, respectively.

**Table 1:** Integral electromiography (EMG) activity (μVs) during rest and MVC before and after temporary bite-raising

	Before temporary bite-raising		After temporary bite-raising		Mean difference (95% CI)	p-value
	Mean	S.D.	Mean	S.D.		
Masseter muscle						
Rest	117.3	54.4	109.9	33.1	7.5 (−15.5 to 30.5)	0.673
MVC	532.3	253.6	331.1	155.8	201.2 (120.8 to 281.6)	0.000*
Temporalis muscle						
Rest	222.7	118.5	185.9	88.5	36.8 (−13.1 to 86.7)	0.262
MVC	467.7	231.7	315.9	104.9	151.8 (66.6 to 237.0)	0.001*

CI: confidence interval; MVC: maximum voluntary clench

* Significant difference (p<0.05, paired t-test)

**Table 2:** Normalized electromiography (EMG) activity (%) during rest and MVC before and after temporary bite-raising

	Before temporary bite-raising		After temporary bite-raising		Mean difference (95% CI)	p-value
	Mean	S.D.	Mean	S.D.		
Masseter muscle						
Rest	24.4	16.8	23.2	13.8	1.2 (−2.8 to 5.1)	0.572
MVC	104.0	22.4	73.8	30.7	30.2 (18.4 to 42.0)	0.000*
Temporalis muscle						
Rest	38.8	25.5	35.8	17.2	3.0 (−5.4 to 11.4)	0.704
MVC	109.0	28.6	76.5	36.6	32.4 (17.3 to 47.5)	0.000**

CI: confidence interval; MVC: maximum voluntary clench

* Significant difference (p<0.05, paired t-test)

** Significant difference (p<0.05, Wilcoxon Signed Ranks Test)

**Figure 2:**
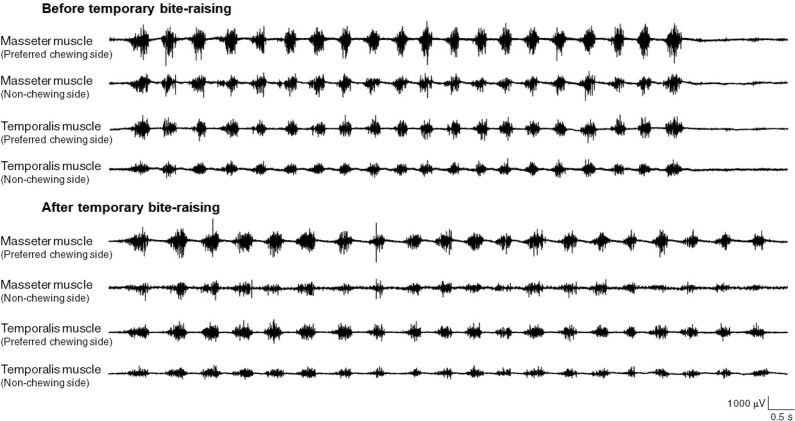
Representative masseter and temporalis muscle electromiography (EMGs)during mastication (test C) before and after temporary bite-raising

Representative EMG recordings for the superficial masseter and anterior temporalis muscles during mastication are shown in Figure 2. The maximum voltage in both muscles was significantly reduced on both the preferred chewing side and non-chewing side after temporary bite-raising (Table 3). The effect size of the maximum voltage for the superficial masseter muscle on the preferred chewing side and the non-chewing side was 72.8 μV and 78.3 μV, respectively. The effect size of the maximum voltage for the anterior temporalis muscle on the preferred chewing side and the non-chewing side was 62.2 μV and 63.3 μV, respectively. However, no significant difference was found for its duration (Table 4).

**Table 3:** Maximum voltage (μV) of the identified burst during mastication before and after temporary bite-raising

	Before temporary bite-raising		After temporary bite-raising		Mean difference (95% CI)	p-value
	Mean	S.D.	Mean	S.D.		
Masseter muscle						
Preferred chewing side	359.0	189.4	231.1	113.3	127.9 (76.6 to 179.3)	0.000*
Non-chewing side	254.1	189.5	160.4	101.7	93.6 (38.4 to 148.9)	0.000**
Temporalis muscle						
Preferred chewing side	183.1	133.1	135.4	95.9	47.7 (3.8 to 91.6)	0.033**
Non-chewing side	145.2	122.5	72.5	56.8	72.7 (28.0 to 117.4)	0.001**

CI: confidence interval

* Significant difference (p<0.05, paired t-test)

** Significant difference (p<0.05, Wilcoxon Signed Ranks Test)

**Table 4:** Duration (ms) of the identified burst during mastication before and after temporary bite-raising

	Before temporary bite-raising		After temporary bite-raising		Mean difference (95% CI)	p-value
	Mean	S.D.	Mean	S.D.		
Masseter muscle						
Preferred chewing side	410.3	111.5	444.4	101.9	-34.1 (−80.1 to 11.9)	0.141
Non-chewing side	393.0	122.3	443.6	129.9	-50.6 (−110.3 to 9.1)	0.094
Temporalis muscle						
Preferred chewing side	427.7	129.8	424.3	137.4	3.4 (−49.5 to 56.3)	0.897
Non-chewing side	392.2	105.2	365.4	120.5	26.8 (−19.8 to 73.4)	0.249

CI: confidence interval

## Discussion

This study investigated the immediate effect of temporary bite-raising using light-cured orthodontic band cement on the occlusal surface of the upper first molars on the EMG activity of the superficial masseter and anterior temporalis muscles. We found that there was no change in EMG activity at rest, however, EMG activity was reduced during MCV and chewing. Based on these results, the null hypothesis was not rejected at rest, but it was rejected during MVC and chewing.

Except for sex, the factors affecting masticatory muscle EMG activity, such as age, facial type, malocclusion, and the EMG recording period, were controlled in this study^5^ ^,^ ^25^. Men and women were not on even numbers in our study. However, the paired t-test or Wilcoxon Signed-Ranks Test was used for within-subject and within-muscle comparisons, thus, the variable EMG responses between participants were eliminated.

The difference was not significant on EMG activity for both muscles at rest, despite being reduced immediately after temporary bite-raising. The cause for this could be the opening distance chosen for this study, set at 2.5–3 mm, which is close to the physiological rest position and is usually adequate for treating orthodontic patients. However, several studies found that increasing the occlusal vertical dimension by ≥3 mm by other means affects the EMG activity of the superficial masseter and anterior temporalis muscles^4^ ^,^ ^11^. Therefore, the EMG response to bite-raising by this method at greater opening distances should be further investigated.

The EMG activity of both muscles during MVC decreased significantly after temporary bite-raising. As reported by Jimenez^10^ (1987), if the occlusion does not result in mandible stability, the jaw-closing muscles will contribute to the stabilization by reducing its EMG activity to avoid damage to other structures. There were only two occlusal contact areas after temporary bite-raising on this study. Despite the adjustments on bite-raising to have even and simultaneous contact on both sides, it might not be sufficient to produce mandible stability. This may have resulted in reduced EMG activity^10^. Ferrario, et al.^7^ (2002) found that the number of occlusal contacts and masseter and anterior temporalis muscle activity during MVC was significantly related in young adults. This may explain why the participants of this study had lower EMG activity during MVC after temporary bite-raising, when compared with the values before temporary bite-raising.

Our results are similar those of Dahlstrom and Haraldson^6^ (1989), who investigated the superficial masseter and anterior temporalis muscles EMG activity using bite plates and suggested that the reduced EMG activity they observed was probably due to the smaller occlusal contacts on the bite plates. Chandu, et al.^4^ (2004) reported similar results with interocclusal appliances that were constructed by pressure forming a laminate base and adding posterior acrylic bite blocks to increase the vertical dimension. However, Wang, et al.^24^ (2013) reported an association between jaw muscle EMG activity and bite force during occluding movement where greater EMG activity was associated with a greater bite force. The limited occlusal contact area results in an uneven distribution of the occlusal force. Thus, the decreased EMG activity in our study might result from a reduced bite force by participants afraid of damaging their teeth. However, we did not evaluate bite force, thus we cannot conclude that the decreased EMG activity was due to reduced bite force. Further investigation including bite force measurements may assist in defining the cause of the reduced EMG activity during MVC after bite-raising with this method. The decreased EMG activity after bite-opening was greater for the superficial masseter compared to the anterior temporalis, which is consistent with the study of Koc, et al.^11^ (2012). This may result from the different muscle locations and orientations, resulting in these muscles being physically affected by changes in jaw position in a different manner. We do not know of any other studies comparing the effects on masticatory muscle EMG activity during mastication after having temporary bite-raising by adding light-cured orthodontic band cement on the occlusal surfaces of upper posterior molars. Thus, comparing our results to previous studies is difficult. Since bite-raising by this method caused a reduced occlusal contact area, our results showed that the superficial masseter and anterior temporalis muscles maximum voltage on the preferred chewing side was significantly reduced after temporary bite-raising. This is consistent with the study of Tomonari, et al.^22^ (2014) who found lower EMG activity for both muscles during chewing on the preferred chewing side of subjects with reduced occlusal surface contacts. The maximum voltage for both muscles was significantly reduced on the non-chewing side after temporary bite-raising. This could be the result from a protective mechanism to control jaw balance, since chewing on one side could cause the mandible to deform and/or tilt around the sagittal axis^13^. The increase on interocclusal distance would cause similar muscle activity on both sides, which might lift the mandible on the non-chewing side, causing excessive temporomandibular joint loading.

The short period of investigation is a limitation of this study. Habituation should be considered when evaluating the effect of bite-raising, since muscle physiology and function may adapt if longer observation periods are allowed. In animal experiments, bite-raising for 2 weeks significantly reduced masseter muscle spindle sensitivity; however, no significant differences were found between control and after more than 6 weeks of bite-raising^26^. However, a long-term investigation with healthy subjects was not possible in our study model due to ethical standards. Therefore, to investigate the precise effect of bite-raising by this method on masticatory muscle activity, a future clinical study should evaluate subjects over an extended period.

## Conclusion

Our results revealed that temporary bite-raising by placing orthodontic band cement on the occlusal surface of the upper first molars had no immediate effect on EMG activity at rest, however, superficial masseter and anterior temporalis muscles EMG activity reduced during MVC and mastication. This information is useful for orthodontists to inform their patients about what will happen to their masticatory muscle activity when this bite-raising method is used. Furthermore, the effects of this type of bite-raising on more clinical-related aspects, such as masticatory performance and masticatory ability should be investigated in future studies.
